# On identity: from a philosophical point of view

**DOI:** 10.1186/1753-2000-7-29

**Published:** 2013-07-31

**Authors:** Daniel Sollberger

**Affiliations:** 1 Psychiatric University Hospital Basel, Wilhelm Klein-Strasse 27, CH – 4012, Basel, Switzerland

**Keywords:** Identity, Individuality, Self, Self-sameness, Identity disturbance, Identity diffusion, Borderline personality disorder

## Abstract

**Background:**

The term “identity” has a much longer tradition in Western philosophy than in psychology. However, the philosophical discourse addresses very different meanings of the term, which should be distinguished to avoid misunderstandings, but also to sharpen the key meanings of the term in psychological contexts. These crucial points in the philosophical concepts of identity in the sense of singularity, individuality, or self-sameness may structure the ongoing discussion on identity in psychiatric diagnoses (as in DSM-5, *Child Adolesc Psychiatry Ment,* this issue, 2013), in psychology, psychoanalysis, but also neuroscience and neurophilosophy (*Child Adolesc Psychiatry Ment,* this issue, 2013).

**Method:**

The concept of identity is subjected to a systematic philosophical analysis following some milestones in its history to provide a background for recent discussions on identity in psychiatry and psychology.

**Results:**

The article focuses first on the philosophical core distinctions of identity in the different meanings to be addressed, second, briefly on some of the diverse psychological histories of the concept in the second half of the 20th century. Finally some reflections are presented on borderline personality disorder, considered as a mental disorder with a disturbance or diffusion of identity as core feature, and briefly on a newly developed instrument assessing identity development and identity diffusion in adolescence, the AIDA that is also presented in the special issue of this journal (*Child Adolesc Psychiatry Ment,* this issue, 2013).

**Conclusion:**

As a conclusion, different points of view concerning identity are summarized in respect to treatment planning, and different levels of description of identity in phenomenology, philosophy of mind, cognitive science, and social science and personality psychology are outlined.

## Background

The term “identity” has a long tradition in Western philosophy and much shorter antecedents in psychology and social psychology. In the last six decades, since E. H. Erikson made his path breaking contributions to psychoanalytic theory and character pathology, elevating the term to a theoretical concept, “identity” has been given many interpretations. Nowadays, in a late modern society we see the difficulties that are related to the ambiguity of the term “identity”. Nevertheless, we realize that it cannot be abandoned but is needed to understand both the successful and the unsuccessful processes of psychological development of children, particularly adolescents, as well as of adults in the so-called “emerging adulthood”. We are confronted with questions on identity in a world, where flexibility is seen as a virtue and accelerating change pervades society, in the vocational world, in families, relationships, and in the biographies of individuals. Thus, beside one of the fundamental questions, like “Who do I want to be, which kind of person and personality?” we are basically challenged by the question “How come that I feel like the same person in my whole life, although many and very crucial things changed and will change, like my age and life cycles, marital status, my friendships, occupation, residence, political engagement, my religious beliefs, and social values? What enables me to feel being the same ‘I’, the same ‘self’, or ‘person’ in all the different roles, that I have to play, with all my different qualities, in the changing course of world events and my biography?”

## Method

The concept of identity is subjected to a philosophical analysis in focusing some core conceptual distinction in the history of the term and its meaning throughout the history of Western philosophy. This provides a basis for a further discussion of the modern psychological and psychopathological concepts of identity and identity diffusion in psychology and psychiatry, particularly in the diagnosis of borderline personality disorders. On this background some comments on the new instrument AIDA (Assessment of Identity Development in Adolescence) are made.

## Results and discussion

### Identity in philosophy: some systematic distinctions

In philosophy “identity” is a predicate, which functions as an identifier, i.e. a marker that distinguishes and differentiates one object from another object. Thus, identity in this sense focuses on the uniqueness of the concerned object. Plato firstly made the distinction between “is” as a copula in a phrase and the identifying “is”; thus, Aristotle distinguished identity in its numeric meaning as equivalence from an identifier that defines an object as an individual. The problem of identity became a problem of substance throughout the history of philosophy in the efforts to define the principle of individuation. Leibniz in his *Discourse on Metaphysics* (Section 9) [1], p. 308 summarized this principle in a mathematical law: it states that no two distinct things exactly resemble each other; otherwise they would be “indiscernibles” and therefore one thing. In other words: two things are indistinguishable and in fact one single thing, if everything that truly can be said of the one may be said of the other as well. So, they become replaceable salva veritate (truth preserving) in any other possible context and under any other conditions.

#### Quantitative identity

One meaning of the term “identity” goes back to the Greek term “atomon”. However, “atomon” signifies “indivisible” in a primary etymological sense; identity in the sense of individuality is a secondary sense. Nevertheless, ‘individuality’ became first the topic of the philosophical mainstream discourse. That is where the conviction of an ineffability and unknowability of individuals is rooted herein. Nothing can be said of an individual than “this one” (‘tode ti’ in Greek), what means that we can identify an individual only by pointing at it, him or her. Leibniz’ contribution to this point in his theory of monads is an attempt to singularize individuals by a complete enumeration of their qualities [2], p. 235. The metaphysical presuppositions of Leibniz’ theory like infinite space and time shall not be discussed here. But, as one can see, the individuality of a singular object is not easily (even principally not) describable in its empirical dimensions. The “I” as such a singular ‘object’ cannot be characterized by a complete enumerations of its characteristics but reduces us to statements about a localization in space and time.

In contrast, Kant argued that individuals couldn’t be specified in terms of a concept of substance as Leibniz attempts to do: We never would come at the individuality of an individual by its complete description. Individuals primarily are objects, i.e. data in perception and as such bound to space and time. Thus, “identity”, in contrast to “existence”, is not a term of ontology but of epistemology. Anyhow, Kant wanted to attribute identity to the unity of an object in a fundamental way, i.e. a priori and incircumventable. In the transcendental ego he laid down such a principle: Being aware of ourselves as thinking subjects we know the subject as being a unified and unique whole (the ‘same’) in all of its different perceptions or thoughts: “The I think must be able to accompany all my representations“ [3], §16, B 131. The Kantian sense of identity is exclusively part of the consciousness of the ego that can be seen as a link to the Anglo-Saxon tradition of empirism in Hobbes, Locke, and Hume.

But, a new problem arises when individuals are identified as singular entities because of their spatio-temporal localization, as it is demanded by empirism: If we refer to an individual by his spatio-temporal localization, thus indicating the individual’s absolute uniqueness, it would be impossible to recognize individuals as the same individuals at different times. This problem becomes highly virulent when it is about personal individuals. Besides, J. Locke (1690) particularly related the human self to memory and, emphasizing this point, argued that a person can be addressed as the same person if she is able to remember previous states of consciousness: a person can “consider itself as itself, the same thinking thing, in different times and places [4], Book II, Chap. 27, 9. If he or she does remember nothing of his or her past her or she “literally has no identity” [5], p. 71. However, numeric identity has to be complemented by substantially meaningful identification, particularly when it is about individual persons [6].

#### Qualitative identity

Thus, identity in the question “Who is it?” intending a merely *numeric-quantitative, precise identification* is transformed into a *qualitative meaning* in the question of “What kind of person or character am I and do I want to be?” Identification is then a matter of classifying something or somebody *as* something respectively somebody; or more technically spoken, as language philosophy does, classifying the individual token (the object as the instance of a concept) by assigning to a type (the concept). This is the usual interpretation of identity in social science. It is a form of “*qualitative identity*” that is specified by detailed, conceptual or substantial attributes: we describe somebody by the particular social roles, which he or she assumes or refuses to assume in his or her action orientation and life praxis, by the ideals and values that matter to him or her, by specific habits, capacities, skills, and biographical experiences [7]. Whether or not this qualitative identity is only a matter of looking at the individual from the perspective of social role, is widely discussed: whereas E. Goffman [8] for example argues that the identity of the I is only a matter of an internal, reflexive perspective of the subject, the German philosopher E. Tugendhat [6] emphasizes that the identification by social roles assumes a commitment by the individual, and depends therefore on an internal view of the individual person from his or her point of view.

Finally, it seems to depend on the epistemological interest: regarding the individual in its qualitative identity from a theoretical point of view as an objectifiable datum it is of no or only little interest in what form and to what extend that individual is committed to the attributed qualities. Whereas from a practical philosophical point of view the individual’s internal attitude to objectifiable social roles crucially matters. Since, this is a question of self-identification: “What kind of person do I want to be?”

The missing link between these two perspectives on the qualitative identity of an individual becomes apparent when we inquire whether an individual identified by social roles (whether intrinsically committed to them or not) can adopt these different roles in different social situations without losing his or her identity. This is probably not a question of identity in the sense of a qualitative identification – and much less one of a numeric one. But, it focuses on the other aspect of identity of individuals: not on the aspect of being different from others but in not being different from oneself – the aspect of “sameness” [2], p. 251 and constancy.

#### Identity as self-sameness

If we focus on this aspect, identity is questioned as a structure or form of an individual’s self-relation and self-conception. Identity in this sense aims at competences and capacities of the individual to communicate, to interact, and to integrate and synthesize different emotional states, social roles, values, beliefs, group identifications etc.. It is exactly the point that Erikson has in mind differentiating role identities from “self-sameness” as the capacity to maintain an inner coherence and continuity:

“The term ‘identity’ expresses such a mutual relation in that it connotes both a persistent sameness within oneself (self-sameness) and a persistent sharing of some kind of essential character with others […] At one time, then, it will to refer to a conscious sense of individual identity; at another to an unconscious striving for a continuity of personal character; at third, as a criterion for the silent doings of ego synthesis; and, finally, as a maintenance of an inner solidarity with a group’s ideals and identity. In some respects the term will appear too colloquial and naïve; in others, vaguely related to existing concepts in psychoanalysis and sociology [9], p. 109.”

The term “ego identity” therefore does not indicate a substance or absolute entity that is independent of interaction, but rather a capacity for an inner coherence of emotional and cognitive states and interaction in social situations as well as an inner continuity over time. Ricoeur contributed a subtle distinction to the discussion in differentiating the aspect of self-sameness, which he called “idem-identity”, and the one of selfhood or self-relatedness, for which he coined the term “ipse-identity” [10]. Identity in the sense of self-sameness is not a fixed result of a process of development, but a dynamic process of continual integration itself, creating continuity in the persistent self-consciousness over space and time. Finally, this meaning of the term “identity” is related to moral philosophical contexts of autonomy and self-determination in responsible decision-making. As the philosopher H. Frankfurt argues, an individual is autonomous and an active agent if he or she has “second order volitions”, i.e. that he or she wants certain desires to be part of his or her will [11], p. 16: “Having an integrated, stable, and coherent identity is an essential precondition for effective second order volitions that stay the same over time” [12], p. 353. Identity in the sense of sameness or constancy over time can be realized as the effort and performance of creating the unity of an autobiographical self (see also [13]): integrating different and sometimes conflicting role identifications means transforming them permanently to a conflict free or less conflicted entity that makes up the unique autobiographic history of our life for which we are responsible. The term “narrative self” or “narrative identity” emphasizes on this higher level identity in the sense that the self is continuous through time and under different conditions and that the person is able to construct a coherent narrative or life story to integrate his or her personal identity. Moreover, the narrative identity is always articulated through concepts (and practices) made available by cultural narratives, i.e. “by religion, society, school, and state, mediated by family, authority figures, peers, friends” [14], p. 20. Therefore, in contrast to a paradigm of cognitive representations the narrative approach entails a shift to a paradigm of social construction, in which the self in its identity, and particularly in its moral development is focused in terms of intersubjectivity: the selves in their identity are embodied, relational, and fundamentally dialogical [15].

The numerous attempts in defining the qualitative identity of a person with sufficient or necessary criteria for personhood such as bodily identity, brain identity, memory, and psychological connectedness or continuity [16] find its limitations, particularly in Anglosaxon debates. The search for such criteria by analytic philosophers is executed from a third person perspective (which focuses on persons as objects or as facts in the world). However, personhood in its identity is not a quality belonging to the owner like blue eyes or impulsiveness, thus, the first person perspective cannot be left out: “Who I am is not a fact about me, but should be phrased in terms of from where I come and what I am up to” [17], and this usually is answered by telling a narrative story. The emphasis on the first person perspective in the phenomenology of the self [18] has become one of the crucial topic in the recent debate on the self in neuroscience and psychiatry and in the progress in linking theories and experimental procedures from psychology to the results of neuroscience [19].

### Identity and identity diffusion in psychological and psychopathological concepts

As mentioned above the history of the concept of “identity” is relatively short in psychology and social psychology compared to its history in philosophy. Whereas the term “identity” or “identity disturbance” is hardly used by S. Freud, it became a core construct in psychoanalytic and psychodynamic theories in the middle of the 20th century.

Earlier, at the end of the 19th century the philosopher and psychologist *William James*[20] made a core distinction between two aspects of identity. Sociology, socio-cognitive sciences, as well as psychoanalysis in their different uses of “identity”, “self-concepts” and “mental representation”, make use of this distinction: The “me” representing the permanence and continuity of the self through the shifting variety of experience constitutes identity across time and is related to how the individual sees the story of his or her past (narrative self-reference), the self, seen as object of perception and reflection. The “I” in contrast, acting in the consciousness of experiences is addressed as an integral part of consciousness, as self-consciousness – or, as modern philosophers name the pre-reflective self-consciousness the “phenomenal” or “minimal self” (for this distinction see also [13]). *G. H. Mead*[21], referring to this core distinction, focused on the interactional and social aspect of the “me” as a part of the self that deals with “society”, i.e. social roles and group identifications, resulting from the experienced interaction and response from others. The term “I” in contrast is intended to represent the socially irreducible spontaneity of the self. From this interactionistical point of view, the identity of individuals is seen as being in need for social recognition, recognizing that humans are fundamentally social beings: “We infer who we are by observing how we are perceived by others and how others react to us” [22], p. 624. Therefore, identity is strongly related on the one hand to the “need for security (the need to belong, to be part of a community and to be attached to others)”, and, on the other hand, to “the desire for freedom (the desire for separation, individuation and autonomy)” [23], p. 30; see also [24], p. 623f.

*Erikson*, who initially made the term “identity” an important concept in his work on psychoanalytic theory and character pathology, referred to W. James by stressing on the importance of the “conscious sense of individual uniqueness”, the “I” in its spontaneous, self-evident acquaintance with itself matching the “unconscious striving for a continuity of experience” [23], p. 208.

The “me” in its continuity results from a developmental process, in which the adolescent at last passes through what Erikson called an “identity crisis” [25] that on the one hand might lead to ego-identity as an “integrated awareness and knowledge *about* oneself” [26], integrating the confirmation of the self by significant others as a core aspect of normal identity. In this conception identity effects an overall synthesis of ego functions as well as a sense of “the solidarity with a group’s ideals” [23], p. 208. Erikson actually puts together all the distinctive philosophical meanings of identity outlined above. Identity in Erikson’s work represents therefore a developed fundamental organizing principle that provides a sense of continuity (“self-sameness”) as well as it structures the differentiation between self and others (“individuality as uniqueness”).

On the other hand, if this process of developing a normal capacity for self-definition fails, then the adolescent doesn’t just pass through a period of “crisis”, in which there is a lack of correspondence between the rapidly changing self-experience of the adolescent and the diverse experiences of him or her by others. Thus, Erikson made the distinction between “identity crisis” and “identity diffusion” [27], where the adolescent experiences an emotional breakdown and conflicts in intimate relationships, over occupational choice, and competition, and becomes increasingly dependent on an increased psychosocial support for self-definition [28].

*J. F. Marcia* as one of Erikson’s main follower focused in his operationalized concept of four different “identity status” on the basic dimensions of exploration and commitment. His orientation was therefore directed more to the question of social adjustment in the identity process than in the work of the other prominent successor of Erikson: O. F. Kernberg. The term “identity diffusion”, as used by Marcia and Kernberg, thus has very different meanings. In Marcia it represents one of the four identity status (achievement, moratorium, foreclosure, identity diffusion), in which both commitment and exploration of the person are at low performance level. The content of the dimensions (gender roles, vocational choice, political preferences, and religious beliefs), to which the goals, values, and beliefs of a person are directed and which the empirical measurements of identity status are based on, are actually core elements of personal identity rather than ego identity that is addressed by Kernberg (see below). In this conceptual scheme one can identify the underlying notion of identity disturbance in borderline personality disorder (BPD) characterized by the DSM-4, which emphasizes commitment and social functioning as fundamental elements of the ego identity [29], p. 651; [30]. Therefore, facing current conditions, Marcia quite rightly discussed the term of identity diffusion later in his work as no longer just a kind of cataclysmic breakdown but maybe an *adaptive* form of identity that has to be positively evaluated: „it is adaptive to be diffuse in a society where commitment is not valued, and, in fact, may be punished“ [31], p. 292. In the terms of the originally proposed DSM-revision the intended content of identity diffusion in this meaning leads to the above-mentioned aspects of self-direction (instability in goals, aspirations, values etc.).

On the other hand, *Kernberg* on his part focused rather on a notion of identity that “provides a psychological structure determining the dynamic organization of character” [32], p. 11. This intra-psychic structure does not contain all of the different aspects of identity but – as an ego identity – it provides to some extent the basis and precondition for at least three further levels of identity such as personal identity, social identity, and collective identity. Ego identity manifests itself “in conscious representations of the self, others, and the world in general, and in identifications with social groups, cultural norms, ideals, and values” [12], p. 346. Kernberg concedes to Erikson’s concept of identity that it already stresses the relevance of the relationship between the self-concept and the concept of a significant other. But, he himself focuses particularly on this background of object relations theory. He defines identity diffusion therefore as “a structural, pathological consolidation of the internalized world of object relations, reflected in a stable lack of integration in the concept of self and significant others” [28], p. 980.

### Identity diffusion in borderline personality disorders

Many researchers and clinicians consider identity diffusion or disturbance to be one of the core diagnostic criteria for borderline personality disorders – despite of its equivocal meaning and different operationalization in empirical research ([12,24,30,33]; Sollberger et al.: Change in identity diffusion and psychopathology in a borderline personality disorder specific TFP-based inpatient treatment, submitted). Kernberg even argues that identity diffusion is “the key anchoring point of the differential diagnosis of milder types of character pathology and neurotic personality organization on the one hand, and severe character pathology and borderline personality on the other” [34]. His concept of borderline personality organization (BPO) reflects the subjective and behavioral consequences of identity diffusion and is regarded as the basic psychopathological syndrome of all severe personality disorders [28].

The common thread of all these concepts is that identity diffusion indicates a lack of differentiated and integrated representations of the self and others, a negative self-image, the lack of long-term goals, and the lack of a sense of continuity in self-perception over time [9,12,24,28,35-39]. As a result, patients experience rapid shifts in the way they view themselves and others, discontinuities and shifts of roles, and a sense of inner emptiness. Moreover, feelings of loss of integration and a sense of (painful) incoherence have been described [40-42]. Westen and Cohen [39] furthermore pointed out a “lack of a coherent life narrative or sense of continuity over time” and – implicitly referring to John Locke’s emphasis on memory in the concept of identity – a “loss of shared memories that help define the self over time”.

In short, following Erikson’s groundwork on identity two careers and different ways of operationalizing the construct of identity diffusion will be outlined in the sequel: the one that concentrates on sociological contexts and emphasizes the importance of commitment and social functioning that results in long-term goals, values, and beliefs; the other that focuses on intra-psychic structure that integrates the concept of self and significant other in personality organization.

### Brief comments on AIDA

#### Philosophical distinctions

On the basis of the considerations made above, I will end by making two remarks on the new instrument AIDA (Assessment of identity development in adolescence), which is presented and discussed in the current special issue of this journal.

What is fundamental and convincing in the theoretical model underlying AIDA is the inherited dichotomy of the construct “identity” [26]: namely, identity in its qualitative meaning answering to the question “Who am I?” and in the sense of self-sameness answering to the question: “Am I the same person (i.e. same I, self, individual) over time and in different situations (continuity) and in my different emotional and cognitive states (coherence)?”

Regarding these two dimensions of identity the one comprises more a subjective, emotional self (the “I” in Mead’s conception), which denotes the aspect of an immediate and intuitive first-person-perspective in all the subjective experiences and inner feelings. The other denotes coherence and continuity in a sense of a self-definition resulting from cognitive functions such as memories and autobiographical memories, self-reflection, but also resulting from motivational states or social role and group identification, which turn the “I” into an identifiable “Me” [21].

Northoff [13] in a more fundamental, philosophical way distinguishes between the “minimal self … that occurs immediately and is always already part of our experience of the world“ (rooting a phenomenal “mineness” and “belongingness”), on the one hand, and the self in its continuity across time and in its other features such as “unity, first-person perspective, and qualia“, on the other hand. He particularly argues that “any experience of the self is part of an experience of the world” as well as “any consciousness of the world goes along with an experience of the self”. Both experiences are inseparable intertwined that this might be a “principal limitation” for experimental investigations of the minimal self^1^.

#### Short philosophical note on neurocognitive research

This is highly relevant in regard of the model of self and identity that underlies research in cognitive neuroscience. Since, considering that the self is not a metaphysical entity (mental substance) but has rather to be comprehended as and replaced by an inner model of self–representation (as e.g. Thomas Metzinger does [43]), this changes the methodological approach to self and identity in a fundamental way. Identity shifts from a philosophical to an empirical research topic and is subjected to cognitive psychology, finally to cognitive neuroscience. Hence, the question raises how all the information of our own body and own brain is processed, i.e. summarized, integrated and coordinated that ultimately an inner model of self-representation reasonably results. This is a matter of different and specific higher-order cognitive functions such as working memory, attention, executive function, semantic and episodic memory etc. that are underpinned by specific brain processes that can be subjected to empirical investigations [13].

However, as we might reasonably question this sceptical tendency to eliminate the notion of self and identity on reductionist grounds, the minimal self, as it is implicitly experienced in consciousness (i.e. consciousness about the world as well as the self being conscious about any world experience), remains specific “self” in contrast to any other experiences. As Legrand and Perrine argue, this self-specificity cannot be “constituted by the integration of contents that are not themselves self-specific” [44], p. 273. In the light of these considerations, cognitive neuroscience should focus on the fundamental difference between neurocognitive processes underlying self-representations (i.e. all representations that have the self as their object and finally result in higher-order cognition such as an “autobiographical self” [45]), on the one hand, and neurocognitive processes that are related to a “phenomenal” or “minimal self”, which is considered as a presupposed self-specific process underlying the former (differentiable) representations (of world and self experiences), on the other hand. Since, self-related or self-directed contents concerning a self-reference effect (SRE), i.e. the fact that stimuli that are related to one’s own self (e.g. a scalpel for a surgeon) show a superiority of memory recall in contrast to those that are not directed to the self [13], may not be confounded with the scope of self-specificity (the I as “representing self” not in its “self-representation”). Thus, neurobiological investigations of the minimal self in its pre-reflective identity should rather encompass studies “of the nonself-directed but self-specific perspective” than such of “self-directed but non-self-specific representations” [44], p. 275.

Identity of a person in terms of self-relatedness of characteristic attributes is focused on a self-as-content. Personality in this sense can be lost, what we experience, for instance, in the dramatic modifications of the identity of persons during the pathological processes of frontotemporal dementia or Alzheimer disease [46]. Personal identity as a (narrative) conception of oneself and as persistence is impaired in patients with dementia insofar as there are gaps in their memory, as they have, as a result of memory loss, personality changes involving, for example, a decrease in self-control. However, can we therefore say, if the gaps in memory and the changes in personality are sufficiently serious, the person has lost his or her identity and self (s. John Locke’s argument above [4])? Or does identity incorporate growth and decline, thus, identity cannot really be lost in a fundamental sense [47]?

Basically, I argue, that Patient’s suffering from dementia at least remain subjects, i.e. remain capable to differentiate themeselves from another person. Identity in the sense of self-specificity that is addressed in the sense of a phenomenal qualia-self in its first-person perspective is not affected by dementia, since: despite the loss of self-relatedness, the capacity to differentiate between self and non-self, i.e. to specify any representation (perception, sensation, feeling, cognition etc.) as *my* representation (“mineness”, s. above), remains, because self-specificity is not constituted “by the integration of contents that are not themselves self-specific” [44], p. 273.

#### Psychological and sociological considerations

In regard to higher-level, i.e. psychological and social processes of identity development in adolescents and particularly in the formation of BPD the question arises: What turns our attention to identity and its diffusion? Is it the consolidation of interests, goals, and values, which develop a degree of stability in child development and adolescence that eventually give us an inner sense of identity, a kind of a self that is emotionally committed to long-term goals and identified with social groups – a kind of biographic self? Is, therefore, identity diffusion in adolescents and young adults a result of a lack of stability in behavior and attitudes, in interests, goals, values, and aspirations?

Or, on contrary, is identity diffusion a manifestation of unstable relationships, and consequently, of unstable inner representations of self- and object relations, which undermine the sense of self-sameness, which BPD patients suffer from? As mentioned above, the DSM-4 describes (implicitly according to Marcia) identity disturbance in BPD as being “characterized by shifting goals, values, and vocational aspirations” [29], p. 651, underscoring commitment and social functioning as fundamental elements of the Ego identity status. In the initially proposed revision of DSM-5, now included in a separate area of section 3 [48], identity becomes a core construct in the diagnosis of BPD. Accordingly, in the diagnostic process first-line differentiations have to be made on impairments in self-functioning as a main characteristic of personality disorders. They are conceived as impairments in “*identity”*, or they pertain to aspects of “*self-direction”* (instability in goals, aspirations, values etc.). How do these two disturbances in *identity* (in the sense of self-sameness, the “I”) and in *self-direction* (in the sense of qualitative, ‘social’ identity, “me”) interfere? Does continuity in these mentioned ‘social’ terms underscore a coherent, self-reflected, by inner motives guided self (“I”)^2^?

Focusing on the interference of “identity” and “self-direction” (as originally proposed for the DSM-5) we can ask the other way round, what can better be demonstrated in terms of identity diffusion. Wilkinson-Ryan and Westen [42] pointed out that identity diffusion seems rather to manifest itself in specific fundamental factors such as “painful incoherence” or “inconsistency” than in a “lack of commitment”, which in fact they show to be the weakest of four factors in predicting BPD: “painful incoherence”, “inconsistency of beliefs and actions”, “role absorption” and “lack of commitment” (n.b. a fundamental element of Marcia’s conception of identity). Thus, in case of identity disturbance: Is there primarily incoherence in the sense of inconsistency and particularly *painful* incoherence (of which BPD patients might differently be aware of and differently concerned about)?

Again the question, how do the two basic dimensions interfere?

To come to my second point: The German philosopher and sociologist Georg Simmel wrote in 1900 in his book “Philosophy of Money”:

“The lack of something definite in the center of the soul impels us to search for momentary satisfaction in ever-new stimulations, sensations and external activities. Thus it is that we become entangled in the instability and helplessness that manifests itself as the tumult of metropolis, as the mania for travelling, as the wild pursuit of competition and as the typically modern disloyalty with regard to taste, style, opinions and personal relationships [49], p. 484.”

It seems that already at the beginning of the 20th century the problem of identity came up, which is considered at the end of that century to be one of the main topics of late-modern Western societies. It has widely been recognized, on the one hand, that these societies show high rates of social change in values and norms. Desynchronizing and uncoupling processes in family formation, vocational education, employment, and retirement as well as falling in love, getting married and having children have been described [50,51]. Topoi of a lifelong learning and education process emerged. Therefore, particularly adolescents experience a lack of stability in orientation concerning these values, norms, and long-term-goals in essential dimensions of life such as family, religion, morals, vocational orientation, politics, social and national affiliation, but also sexual orientation, sexuality and gender. As a consequence, they are challenged by the task “to forge a personal identity without being able to rely on models from previous generations” [52], p. 90.

On the other hand, identity turns into a sort of “do-it-yourself-project” with a primary task for the “self-made identities” to be “ready to grasp many chances and (…) to adjust to changing necessities” [9], p. 99. Many theoreticians argued about that, like for example Anthony Giddens [53] with his term of an “embeddedness” of the self that he describes as dissolved and dismembered, with an embedded identity; or Richard Sennett, who describes a “corrosion of character” [54] caused by a flexibility pervasive in the restless dynamics of late-modern culture.

We therefore could ask, somewhat provocatively, whether men and women of the 20th century suffer from or enjoy a kind of identity diffusion. Notably, this is what Marcia aims to describe in his conception of identity diffusion as an *adaptive* form of identity under postmodern conditions. Do we not have to reflect on the social changes mentioned above in defining new conceptions of individual identity and its disturbances see [38]? Does identity diffusion, maybe even the borderline syndrome reflect “problems and discourses of late modern culture” [24], p. 636ff.? How do psychology (regarding individual dynamics) and sociology (referring to social changes) challenge an integrated and coherent conception of identity and self – in respect to the fundamental philosophical basis? How do sociological descriptions of ‘late modern man’ impact our conception of the “psychic apparatus”, the structure of personality with its different parts and its conscious and unconscious dynamics and conflicts [55]?

## Conclusion

Assessing “identity”, particularly in contexts of psychopathological developments of individual persons, requires both systematically reflecting on the fundamental philosophical background of the term “identity” and a broad scope of different considerations in regard of neurocognitive research, of psychological and psychopathological phenomenology as well as of sociological developments under contemporary cultural and societal conditions.

To summarize, we have to keep in mind different levels of description in regard of definitions of the term “identity”, in regard of methodological approach investigating “identity” as well as in regard of the person in her identity that is addressed in psychotherapy. It depends on the point of view, which part of identity respectively identity diffusion is focused in research and (clinical) practice. Therefore, in respect of the concept and the diagnostics of emerging personality disorders in adolescence, in which the AIDA is engaged, the factors and mechanisms that lead to identity disturbance have to be considered as multifaceted, complex, and concerning different aspects of identity. Hence, these aspects of identity have to be addressed in planning accurate treatments and in deciding the focus of (psycho-)therapeutic interventions.

From a *sociological* point of view, that is concerned about collective or cultural, and social identity, the societal changes, conceived e.g. as individualization and globalization with an increased mobility and flexibility in professional as well as private, familial relations [54], might impact the development of identity because of the lack of stable models for the adolescents in terms of attitudes, interests, beliefs, and goals. And that might interfere with the development of role identity, e.g. the social adaption of an adolescent in his or her social behavior and moral development.

From a *psychological* point of view, that is concerned about social and personal identity, one might raise the question whether these processes of modernization result in increased challenges for individuals regulating intergenerational, interpersonal and intra-psychic relations: thus, the regulation of care taking and disallowance, caring and separation in parenthood, aggression and rivalry progressively rest on the psychosocial competences of individuals in regard to the lack of traditional collective guidelines.

From a *psychodynamic* point of view, which is concerned about ego identity, on might ask whether intra-psychic conflicts that are related to the human condition (such as oedipal conflicts, experiences of privation or desire) remain “stable” despite of the mentioned cultural changes. Thus, individuals have to pass through crucial phases to develop a personality with its subjective interiority and its sense of identity. Therefore, disturbances in this development of identity concern difficulties in coping with these *inter*subjective as well as *intra*-psychic conflicts.

From a *neurocognitive* point of view identity is addressed in a rather basic sense by differentiating higher-order cognitive functions such as working memory or executive functions, which finally concern self-representing contents, on the one hand, and self-specificity, i.e. the acquaintance of a minimal self, which comprehends a sense of “mineness”, on the other hand. Evidently, the assumption that we can directly link concrete personality traits and underlying, neurobiological mechanisms has to be carfully evaluated. Since, subjective intentionality of behavior is assumed to function on two levels of organismic organization: “a basic neurobiological one, and a derived, secondary, symbolic or psychological one that (…) in turn may influence the functioning of the underlying neurobiological structures” [56], p. 237.

Therefore, *philosophically* we are challenged in defining the complexity of different perspectives in their convergence respectively divergence and incompatibility. It is the question of how to (re-)construct a model of the self in its identity [19] that integrates the perspective of:

• phenomenology, which is concerned about the the essence, content and feel of a mental state, and, concerning identity, the self as implicitly, tacitly, and immediately experienced in consciousness;

• philosophy of mind, which focuses on the logical connection and systematization of our knowledge of mind;

• cognitive science, which designs models of how the mind works as basis for further empirical research, in regard to identity particularly in terms of self-relatedness and self-specificity;

• social science as well as personality psychology, which is concerned with how people regard themeselves, with their different roles in society and the interaction between both.

A philosophically reflected integration of these different perspectives in a conceptual framework will provide a basis for empirical research as well as clinical practice. Furthermore, it will help to distinguish adaptive forms of identity development that our adolescents *and* patients may reveal, and pathological forms in social interaction, in self-related representations, intra-psychic conflicts as well as in their neurobiological correlates – perspectives that finally may provide the key anchoring point of psychotherapeutic (and other) treatments.

## Endnotes

^1^ The question connotes Kant’s reasoning of the transcendental ego: Do I must have a sense of “mineness”, that means that I know what it means that mental states, in which I am, are mine, thus I can refer them to me? Or, must a sort of continuity first has to be developed, so that I know me as the same I as before at that time in another temporal and mental state, to be able to have a feeling of what it means, that cognitions, affective states and so one are mine [57]?

^2^ Probably this more sociologically accented question goes parallel to the chicken-and-egg-problem of the direction of causality in cognitive theories that assume that emotional consistency and predictability over time and across similar situations are a prerequisite for the development of a stable sense of identity; whereas, in contrary, psychoanalytic theories regard the mature personal identity as an important fundament on which cognitive, affective and interpersonal functions are based on.

## Competing interests

The author declares that he has no competing interests.

## Author information

The author has a PhD in philosophy and is leading the department of psychotherapy in the Psychiatric University Hospital Basel as a MD.

## References

[B1] Leibniz GW Loemker L Discourse on Metaphysics Philosophical Papers and Letters 1969 2 Dordrecht: D. Reidel

[B2] Angehrn E Geschichte und Identität 1985 Berlin: Walter de Gruyter

[B3] Kant I Guyer P, Wood A Critique of pure reason 1999 Cambridge: University Press

[B4] Locke J An essay concerning human understanding (1690) 1894 http://www.ilt.columbia.edu/publications/Projects/digitexts/locke/understanding/chapter0227.html [accessed April 17, 2013]

[B5] Kihlstrom JF Beer JS Klein SB Leary MR, Tangney JP Self and identity as memory Handbook of self and identity 2003 New York: Guilford 68 90

[B6] Tugendhat E Selbstbewusstsein und Selbstbestimmung 1979 Frankfurt am Main: Suhrkamp

[B7] Straub J Identitätstheorie im Übergang? Soziologische Literatur Rundschau 1991 23 56 57

[B8] Goffman E Stigma: Notes on the Management of Spoiled Identity 1963 New Jersey: Prentice-Hall

[B9] Erikson EH Identity and the cycle of life. (1st ed. 1959) 1980 New York: Norton & Co.

[B10] Ricoeur P Oneself as another 1992 Chicago (transl. by K. Blamey): The University of Chicago Press

[B11] Frankfurt H The importance what we care about 1988 Cambridge: Cambridge University Press

[B12] Jørgensen CR Invited essay: Identity and borderline personality disorder J Pers Disord 2010 24 344 364 10.1521/pedi.2010.24.3.344 20545499

[B13] Northoff G Brain and Self – A neurophilosophical account Child Adolesc Psychiatry Ment Health(this issue) 10.1186/1753-2000-7-28 PMC3734106 23902725

[B14] Appiah KA The ethics of identity 2005 Princeton: Princeton University Press

[B15] Day JM Tappan MB The narrative approach to moral development: From the epistemic subject to dialogical selves Hum Dev 1996 39 67 82 10.1159/000278410

[B16] Nooan HW Personal identity 1990 London: Routledge

[B17] Glas G Person, personality, self, and identity: A philosophical informed conceptual analysis J Pers Dis 2006 20 126 138 10.1521/pedi.2006.20.2.126 16643117

[B18] Zahavi D Kircher D, David A Phenomenology of the self The self in neuroscience and psychiatry 2003 Cambridge: Dambridge University Press 56 75

[B19] Kircher D David A The self in neuroscience and psychiatry 2003 Cambridge: Dambridge University Press

[B20] James W Principles of Psychology, Volume 1 1890 New York: Henry-Holt & Co

[B21] Mead GH Mind, self, and society 1934 Chicago: University Press of Chicago

[B22] Bauman Z Liquide life 2005 London: Polity

[B23] Erikson EH Identity. Youth and crisis 1968 New York: Norton

[B24] Jørgensen CR Disturbed sense of identity in borderline personality disorder J Pers Disord 2006 20 618 644 10.1521/pedi.2006.20.6.618 17192141

[B25] Erikson EH Growth and crisis of the healthy personality Identity and the life cycle 1950 New York: International University Press 50 100

[B26] Goth K Foelsch P Schlüter-Müller S Birkhölzer M Jung E Pick O Schmeck K Assessment of identity development and identity diffusion in adolescence - Theoretical basis and psychometric properties of the self-report questionnaire AIDA Child Adolesc Psychiatry Ment Health 2012 6 27 10.1186/1753-2000-6-27 22812911 PMC3485126

[B27] Erikson EH The problem of ego identity J Am Psychoanal Assoc 1956 4 56 121 10.1177/000306515600400104 13286157

[B28] Kernberg OF Identity: Recent findings and clinical implications Psychoanal Q 2006 75 969 1004 17094369 10.1002/j.2167-4086.2006.tb00065.x

[B29] American Psychiatric Association (APA) Diagnosic and statistical manual of mental disorders (DSM-IV-TR) 2000 4 Washington: American Psychiatric Publishing

[B30] Sollberger D Gremaud-Heitz D Riemenschneider A Küchenhoff J Dammann G Walter M Associations between identity diffusion, axis II disorder, and psychopathology in inpatients with borderline personality disorder Psychopathology 2012 45 15 21 10.1159/000325104 22123512

[B31] Marcia JE Luszcz MA, Nettelbeck T Identity diffusion differentiated Psychological development across the life-span 1989 North-Holland: Elsevier 289 295

[B32] Clarkin JF Yeomans FE Kernberg O Psychotherapy for Borderline Personality: Focusing on Object Relations 2006 Washington: American Psychiatric Publishing

[B33] Oldham J Clarkin JF Appelbaum A Carr A Kernberg P Lotterman A McGlashan TH A self report instrument for borderline personality organization The Borderline: Current Empirical Research 1985 Washington: American Psychiatric Press 3 18

[B34] Kernberg OF Aggressivity, narcissism, and self-destructiveness in the psychotherapeutic relationship 2004 New Haven: Yale University Press

[B35] Kernberg OF Aggression in Personality Disorders and Perversions 1992 New Haven: Yale University Press

[B36] Marcia JE Ego identity and personality disorders J Pers Disord 2006 20 577 596 10.1521/pedi.2006.20.6.577 17192139

[B37] Bender DS Skodol AE Borderline personality as a self-other representational disturbance J Pers Disord 2007 21 500 517 10.1521/pedi.2007.21.5.500 17953503

[B38] Fuchs T Fragmented selves: Temporality and identity in borderline personality disorder Psychopathology 2007 40 379 487 10.1159/000106468 17652950

[B39] Westen D Cohen RP Segal ZV, Blatt SJ The self in borderline personality disorder: A psychodynamic perspective The Self in Emotional Distress: Cognitive and Psychodynamic Perspectives 1993 New York: Guilford Press 334 368

[B40] Adler G Buie D Aloneness and borderline psychopathology: the possible relevance of child development issues Int J Psychoanal 1979 60 83 96 457345

[B41] Buie D Adler G Definitive treatment of borderline personality Int J Psychoanal Psychother 1982 9 51 87 6759434

[B42] Wilkinson-Ryan T Westen D Identity disturbance in borderline personality disorder: An empirical investigation Am J Psychiatry 2000 157 528 541 10.1176/appi.ajp.157.4.528 10739411

[B43] Metzinger T Being no one 2003 Cambridge/Mass: MIT Press

[B44] Legrand D Perrine R What is self-specific? Theoretical investigations and critical review of neuroimaging results Psychol Rev 2009 116 252 282 19159156 10.1037/a0014172

[B45] Damasio AR The self comes to mind 2010 New York: Viching

[B46] Ruby P Colette F D’Argembeau A Péters F Degueldre C Balteau E Luxen A Maquet P Salmon E Perspective taking to assess self-personality: what's modified in Alzheimer's disease? Neurobiol Aging 2009 30 1637 51 Epub 2008 Feb 6 10.1016/j.neurobiolaging.2007.12.014 18258337

[B47] Lesser AH Hughes JC, Louw SJ, Sabat SR Dementia and personal identity Dementia. Mind, meaning 2006 New York: Oxford University Press 55 61

[B48] American Psychiatric Association DSM-5 facts 2012 http://dsmfacts.org/materials/american-psychiatric-association-board-of-trustees-approves-dsm-5/. [accessed February 21, 2013]

[B49] Simmel G The philosophy of money 1978 Boston: Routledge & Kegan Paul (1st ed. German: Philosophie des Geldes, 1900). Transl. by Bottomore T & Frisby D

[B50] Rosa H Beschleunigung. Die Veränderung der Zeitstrukturen in der Moderne. Frankfurt 2005 Frankfurt a.M: Suhrkamp

[B51] King V King V, Gerisch B Umkämpfte Zeit – Folgen der Beschleunigung in Generationenbeziehungen Zeitgewinn und Selbstverlust. Folgen und Grenzen der Beschleunigung 2009 Frankfurt a.M: Campus 40 62

[B52] Paris J Social factors in the personality disorders: A biopsychosocial etiology and treatment 1996 Cambridge: Cambridge University Press

[B53] Giddens A Modernity and self-identiy: Self and society in the late modern age 1991 London: Polity

[B54] Sennett R The corrosion of character: The personal consequences of work in the new capitalism 1998 New York: Norton

[B55] Dornes M Sollberger D Zum Strukturwandel der Persönlichkeit in der Spätmoderne Übergänge – Konstanz und Veränderung. Psychotherapie & Sozialwissenschaft, Volume 14 2012 17 36

[B56] Kernberg OF Overview and critique of the classification of personality disorders proposed for DSM-V Swiss Arch Neurol Psychiatry 2012 163 234 238

[B57] Sollberger D Über einige "Dunkelheiten" in Kants Kategoriendeduktion Zeitschrift für philosophische Forschung 1994 48 69 8919349661

